# Supplementary material for: Functional characterization of the disease-associated CCL2 rs1024611G-rs13900T haplotype: The role of the RNA-binding protein HuR
Source: bioRxiv. 2025 May 30:2023.10.31.564937. Originally published 2023 Nov 2. Preprint. [Version 2] doi: 10.1101/2023.10.31.564937 (PMC10635030; doi:10.1101/2023.10.31.564937)
Supplement: Supplement 1 — Figure S1. SNP genotyping for rs13900 using TaqMan technology. (A) Real-time multicomponent amplification curves for the TaqMan’s rs13900 assay. Fluorescence signal detected for individuals with rs13900T, rs13900C and rs13900CT genotypes. The blue curve indicates amplification of T allele (alternate/mutant allele) while the green curve represents C allele (wild type). (B) Allelic discrimination plot generated on Quant Studio 12K Flex Real-Time PCR system showing higher signal and better cluster separation for the rs13900 assay. Allele C is plotted on X-axis and the T allele is plotted on Y-axis. Figure S2. Time course of CCL2 mRNA expression in PBMCs. Peripheral blood mononuclear cells (PBMC) were treated with 1 μg lipopolysaccharide (LPS) for 1, 3 or 6 hours. CCL2 mRNA expression was measured by quantitative real-time PCR and normalized to 18S rRNA. Data are presented as mean ± SD of triplicate samples. Figure S3. Validation of HuR overexpression and silencing by Western blotting. (A) A representative western blot showing HuR protein levels in HEK293 cells transfected with either pCMV6-HuR plasmid or HuR-targeting siRNA (HuR-siRNA). (B) Densitometric analysis of Western blot bands. The histogram shows relative intensity of HuR bands in each sample, normalized to β-actin. Error bar represents standard error mean from three independent experiments. *p<0.025, ** p<0.05. Figure S4. Polysomal association of CCL2 mRNA before and after LPS stimulation of macrophages (A) Polysome profile obtained by sucrose gradient centrifugation from macrophages before and after stimulation with LPS (1 μg/mL) for 3 h. The polysome profile shows a shift from monosome to heavier polysome upon LPS stimulation, indicating active translation. (B) The percentage of CCL2 mRNA loading on polysome fraction was calculated using ΔCT method (mean ± SEM, n=4). *p<0.025. Figure S5. Transduction efficiency of pCMV6-HuR. Flow-cytometric analysis of transduction efficiency (Panel A) and the intensity of [file NIHPP2023.10.31.564937v2-supplement-1.pdf]

Figure S1

A

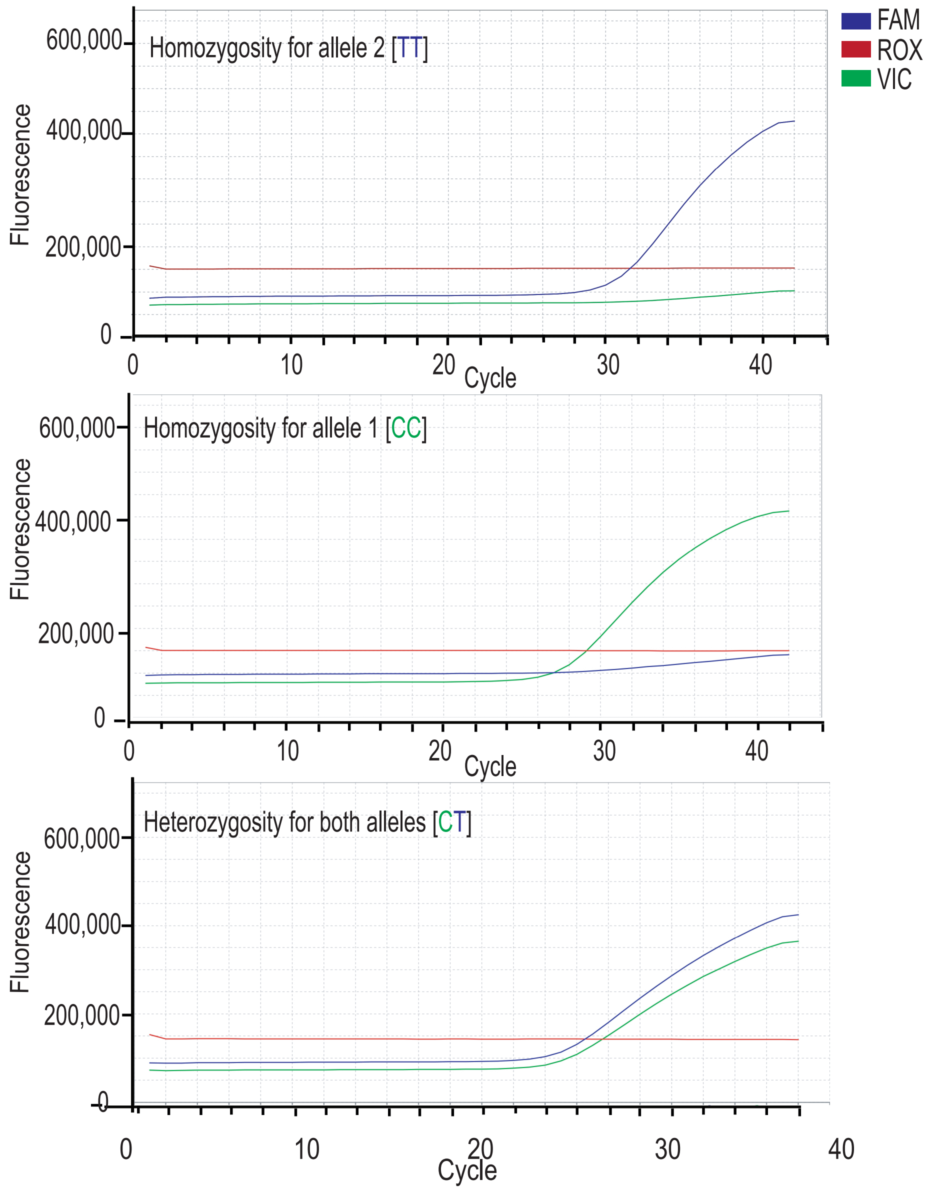

Homozygosity for allele 2 [TT]
FAM
ROX
VIC
Fluorescence
600,000
400,000
200,000
0
0
10
20
30
40
Cycle
Homozygosity for allele 1 [CC]
Heterozygosity for both alleles [CT]

B

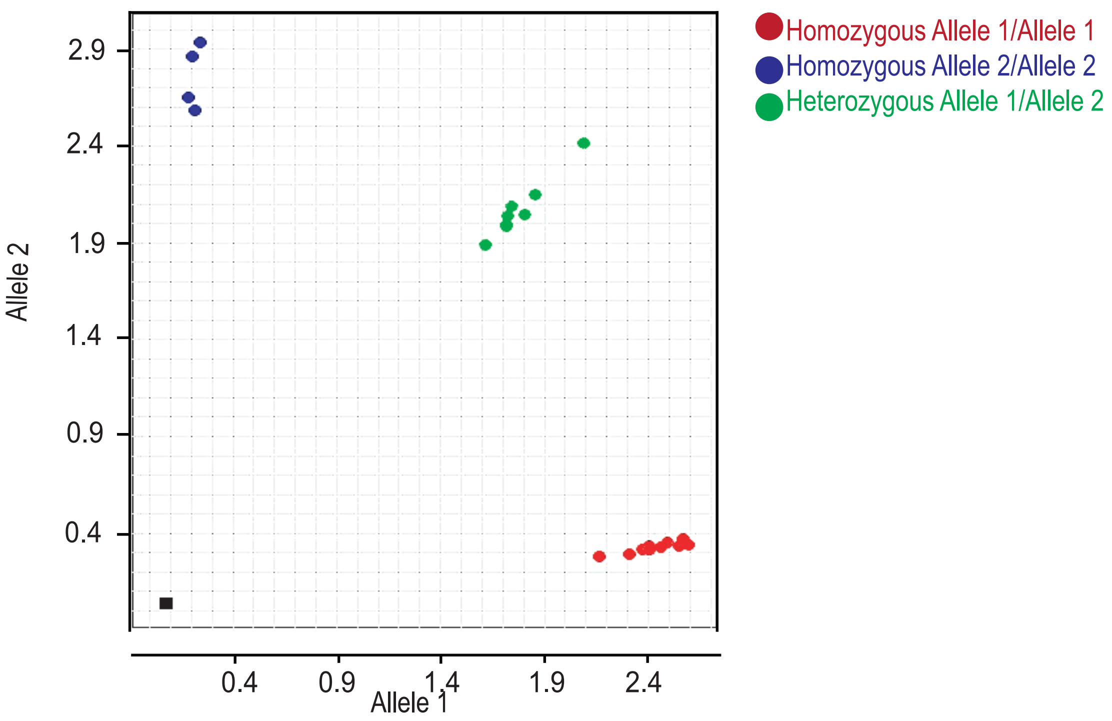

Homozygous Allele 1/Allele 1
Homozygous Allele 2/Allele 2
Heterozygous Allele 1/Allele 2
Allele 2
2.9
2.4
1.9
1.4
0.9
0.4
Allele 1

Figure S2

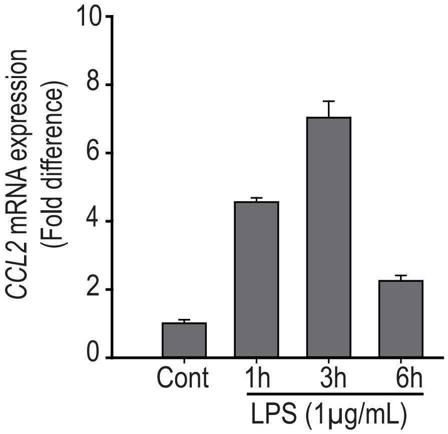

10
8
6
4
2
0
CCL2 mRNA expression
(Fold difference)
Cont
1h
3h
6h
LPS (1μg/mL)

Figure S3

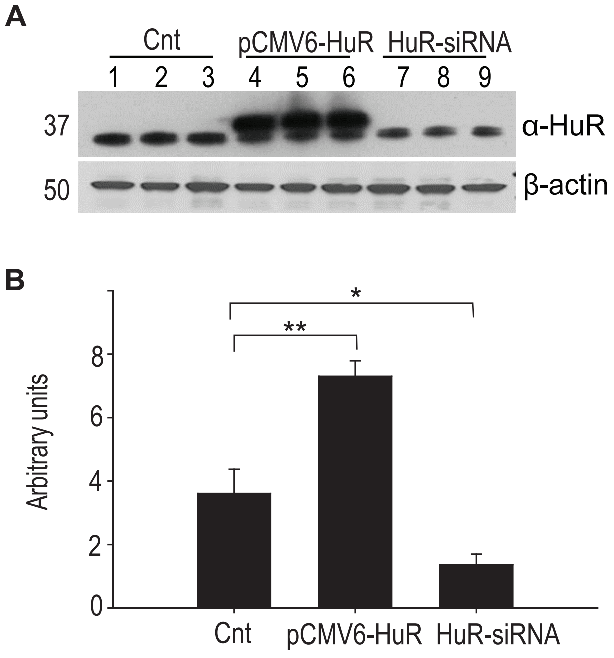
A
Cnt
pCMV6-HuR
HuR-siRNA
1 2 3 4 5 6 7 8 9
37
50
α-HuR
β-actin
B
Arbitrary units
0
2
4
6
8
**
*
Cnt
pCMV6-HuR
HuR-siRNA

Figure S4

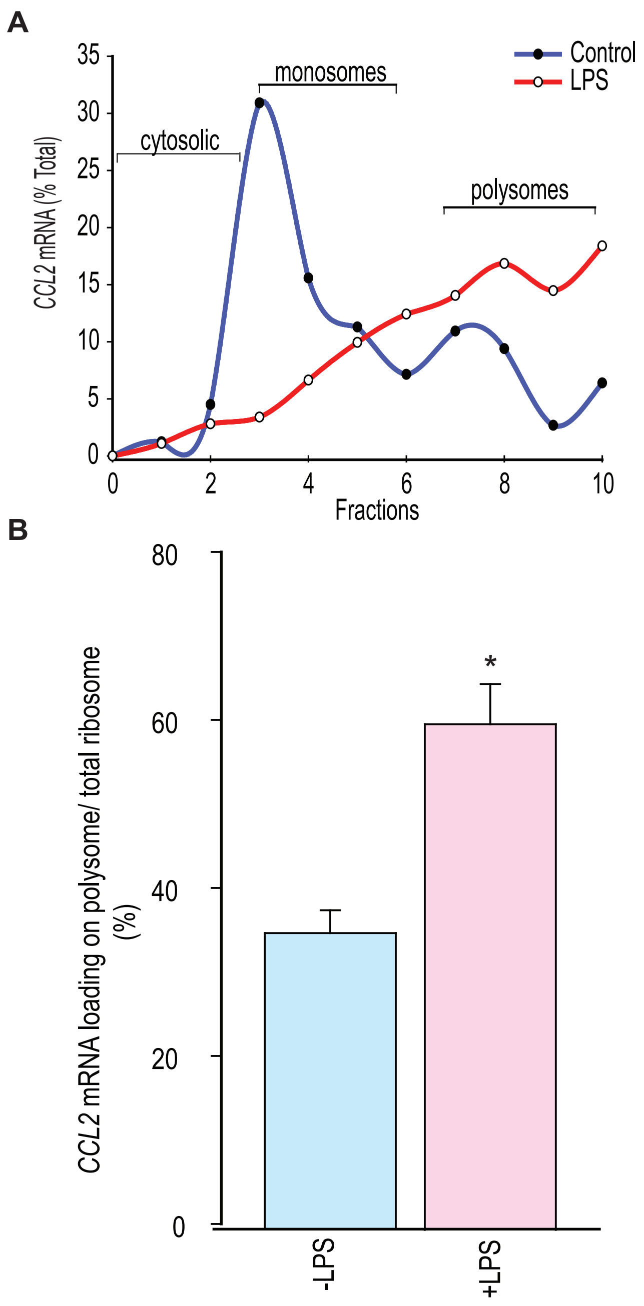
A
Control
LPS
monosomes
cytosolic
polysomes
CCL2 mRNA (% Total)
35
30
25
20
15
10
5
0
0
2
4
6
8
10
Fractions
B
80
60
40
20
0
*
CCL2 mRNA loading on polysome/ total ribosome (%)
-LPS
+LPS

Figure S5

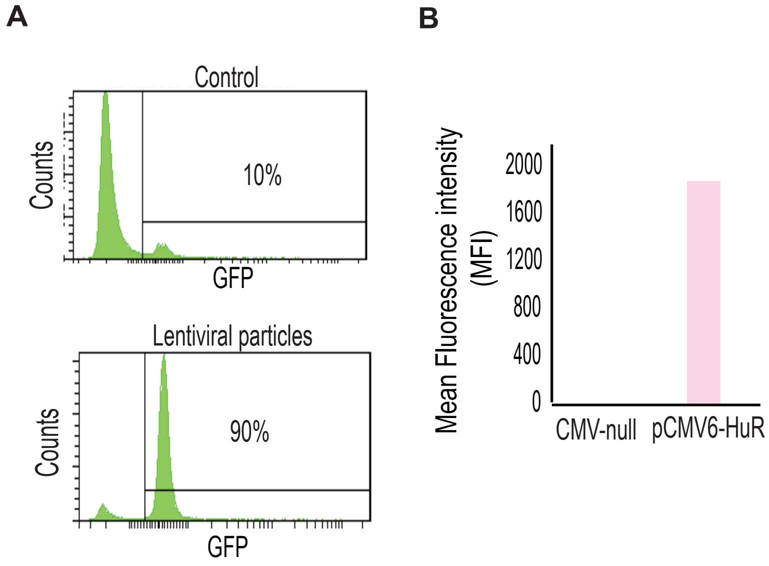
A
B
Control
Counts
10%
GFP
Lentiviral particles
Counts
90%
GFP
Mean Fluorescence intensity
(MFI)
2000
1600
1200
800
400
0
CMV-null
pCMV6-HuR

Figure S6

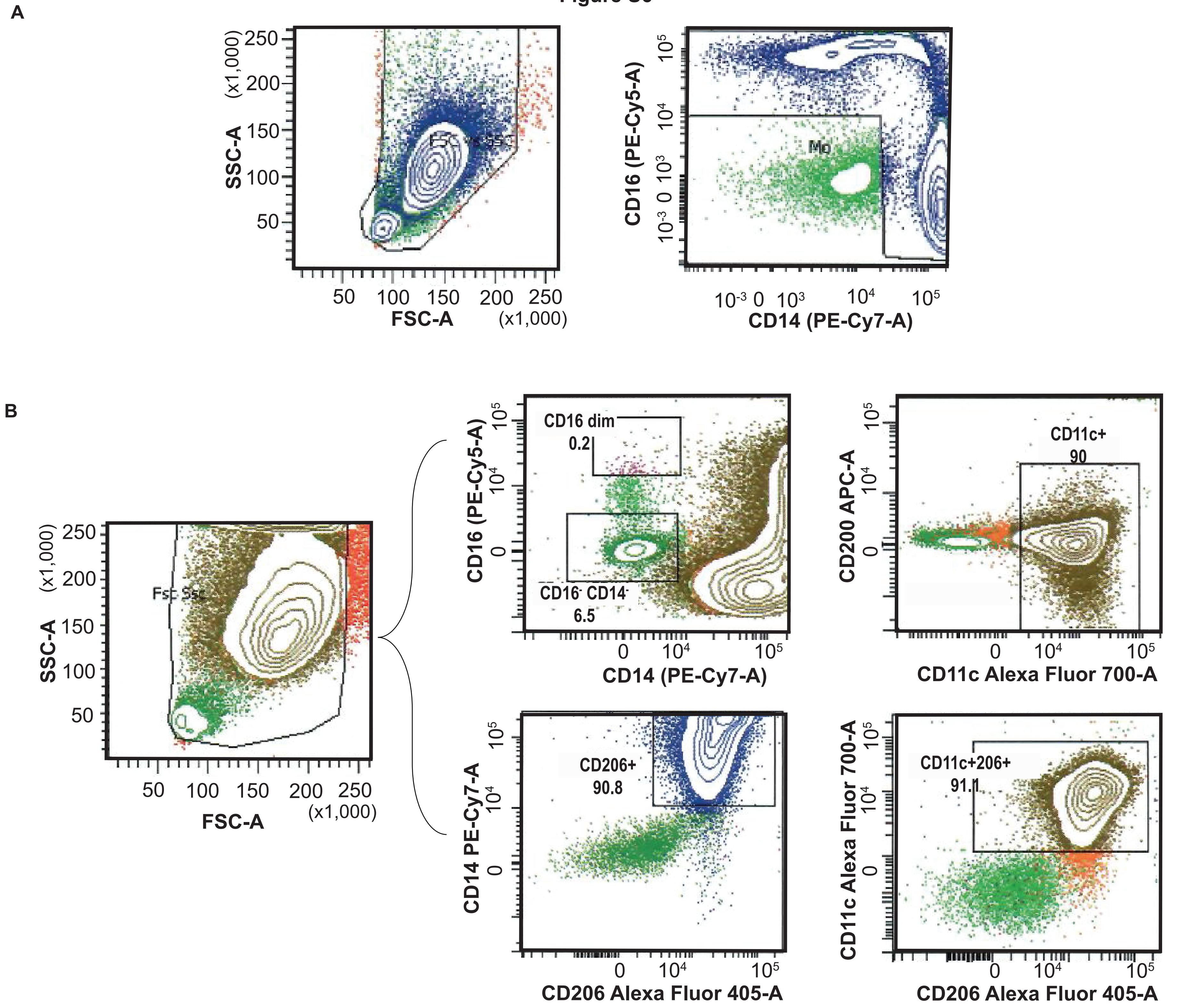
A
SSC-A
(x1,000)
250
200
150
100
50
FSC-A
(x1,000)
50 100 150 200 250
CD16 (PE-Cy5-A)
CD14 (PE-Cy7-A)
Mo
B
Fsc Ssc
CD16 dim
0.2
CD16- CD14-
6.5
CD11c+
90
CD200 APC-A
CD11c Alexa Fluor 700-A
CD206+
90.8
CD14 PE-Cy7-A
CD206 Alexa Fluor 405-A
CD11c+206+
91.1

Figure S7

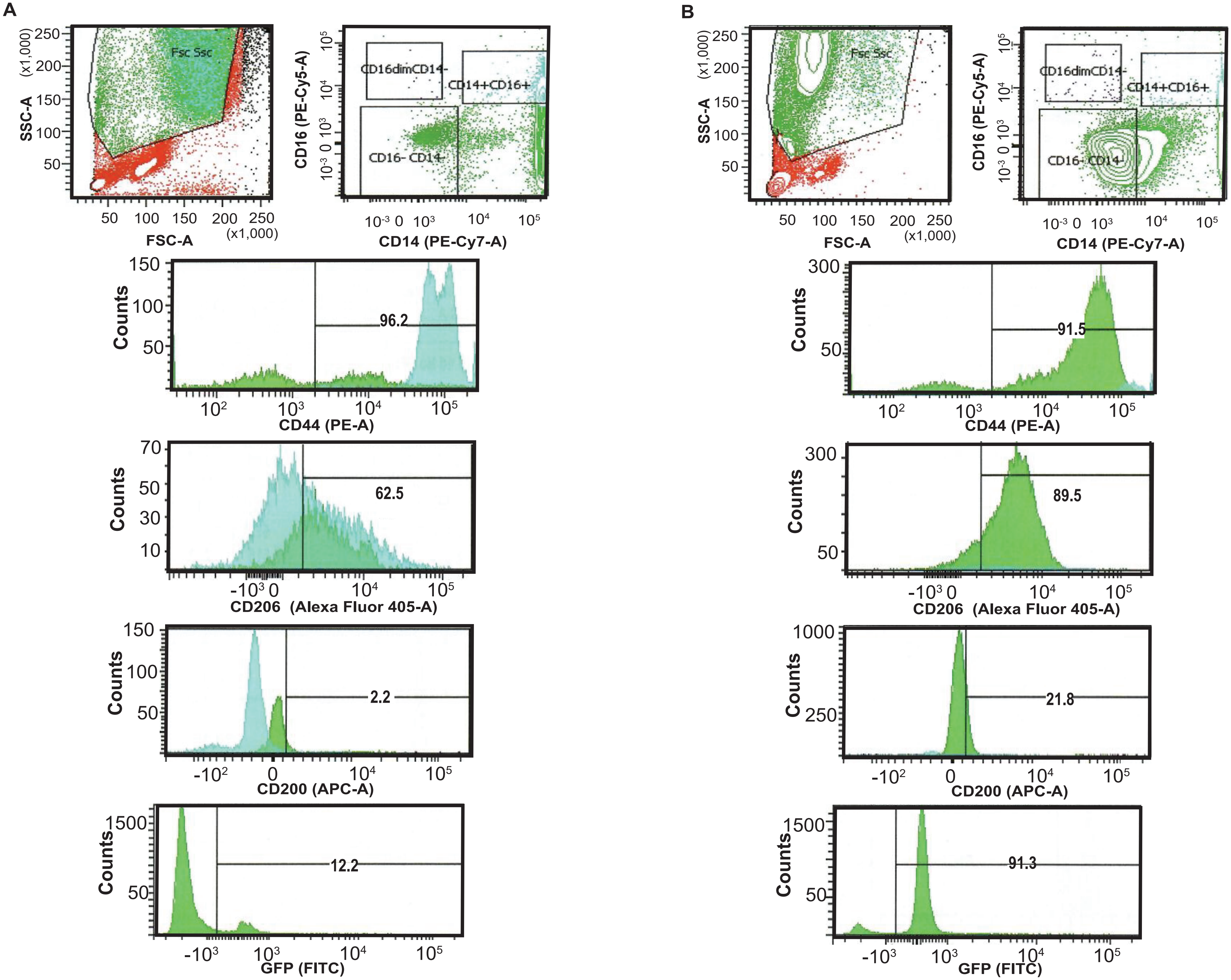

A
B
Fsc Ssc
SSC-A
FSC-A
(x1,000)
CD16dimCD14-
CD14+CD16+
CD16- CD14-
CD16 (PE-Cy5-A)
CD14 (PE-Cy7-A)
Counts
96.2
CD44 (PE-A)
62.5
CD206 (Alexa Fluor 405-A)
2.2
CD200 (APC-A)
12.2
GFP (FITC)
91.5
89.5
21.8
91.3

	rs13900 (n=42)
Reference allele [CC]	18 (42.8%)
Heterozygous [CT]	16 (38.09%)
Alternative allele [TT]	8 (19.04%)
MAF	0.38
Heterozygosity	0.47
No of alleles	84
Hardy Weinberg *P*	0.459

Supplementary Table 1. Allele carriages and allele frequencies of rs13900 in healthy volunteers, MAF, minor allele frequency.

RBP	Tissue type	rs#	Position	Strand	CLIP-seq technology and peak calling method	Score	PhastCons score	PhyloP score	Data accession	Ref strand	Alt strand	SNP position
ELAVL1	HeLa	rs13900	chr17:34256881-34256901	+	PAR-CLIP,Piranha_0.01	11	0.024	0.227	GSE29943,GSM741175	C	T	chr17:34256891-34256892
ELAVL1	HeLa	rs181021073	chr17:34256857-34256876	+	PAR-CLIP,PARalyzer	0.647	0.002	0.14	GSE29943,GSM741173	T	G	chr17:34256868-34256869
ELAVL1	HeLa	rs181021073	chr17:34256857-34256876	+	PAR-CLIP,PARalyzer	0.653	0.002	0.14	GSE29943,GSM741174	T	G	chr17:34256868-34256869
ELAVL1	HeLa	NA	chr17:34257108-34257130	+	PAR-CLIP,PARalyzer	0.623	0.001	-0.256	GSE29943,GSM741173	AT	A	chr17:34257109-34257110
ELAVL1	HeLa	NA	chr17:34257101-34257121	+	PAR-CLIP,Piranha_0.01	9	0	-0.39	GSE29943,GSM741173	AT	A	chr17:34257109-34257110
ELAVL1	HeLa	NA	chr17:34257101-34257121	+	PAR-CLIP,Piranha_0.01	6	0	-0.39	GSE29943,GSM741175	AT	A	chr17:34257109-34257110

Supplementary Table 2. Crosslinking immunoprecipitation (CLIP) analysis of HuR binding sites on the 3'untranslated region (3'UTR) of the *CCL2* gene. Summary of the SNPs located within HuR-binding regions is shown including SNP ID (rs#), genomic coordinates, strand, binding score, conservation scores (PhastCons, PhyloP), dataset accession numbers, alleles, and SNP position within peak region. Data was generated using POSTAR3 using the variation module.

	gDNA	Cytosol	Monosome	Polysome
D-1	0.975806	0.872946	0.873425	1.778429
D-2	1.034091	1.557778	1.441919	1.562622

Supplementary Table 3. Loading of rs13900 alleles to cytosolic, monosomal and polysomal fractions from macrophage extracts prepared from heterozygous donors. T:C ratio > 1 indicates increased levels of the T allele relative to C allele.

S.N	Reagents	Source	Cat.No.	Note
1	rs13900 TaqMan Assay	ThermoFisher Scientific	4351379	C7449810_10*
2	QIAamp DNA Blood Mini Kit	Qiagen	51104	
3	TaqMan Genotyping Master Mix	ThermoFisher Scientific	4371355	
4	Histopaque-1077	Millipore Sigma	10771	
5	Lipopolysaccharides from *E.coli* 0:111:B4	Millipore Sigma	L2630	1µg/mL**
6	EasySep Human Monocyte Isolation Kit	StemCell Technologies	19359	
7	Recombinant Human M-CSF	Peprotech	300-25	50 ng/mL**
8	Pe-Cy 7 Mouse-Anti Human CD14$^+$	BD-Pharmigen	562698	
9	FITC Mouse- Anti Human CD64+	BD-Pharmigen	555522	
10	BV421 Mouse Anti-Human CD206+	BD-Pharmigen	564060	
11	AlexaFluor 700 Mouse-Anti Human CD11c$^+$	BD-Pharmigen	561352	
12	PE CY 5 Mouse Anti-Human CD16+	BD-Pharmigen	555408	
13	RNeasy Plus Mini kit	Qiagen	74134	
14	MultiScribe Reverse Transcriptase	ThermoFisher Scientific	4311235	
15	CCL2 TaqMan Gene Expression Assays	ThermoFisher Scientific	4331182	Hs00736046_m1*
16	18S TaqMan Gene Expression Assays	ThermoFisher Scientific	4331182	Hs03003631_g1*
17	Click-iT-Nascent RNA Kit	Invitrogen	C10365	
18	Actinomycin D	MilliporeSigma	A1410	
19	Superscript Vilo Kit	ThermoFisher Scientific	11754250	
20	AmpliTaq Gold 360 Master Mix	ThermoFisher Scientific	4398881	
21	GeneJET PCR Purification Kit	ThermoFisher Scientific	K0701	
22	ELAVL1 human recombinant protein	OriGene	TP301562	
23	HuR/ELAV1 Antibody	Santa Cruz	sc-5261	
24	hnRNP E1 Antibody (T-18)	Santa Cruz	sc-16504	
25	Normal Goat IgG	Santa Cruz	sc-2028	
26	β Actin Antibody (2A3)	Santa Cruz	sc-517582	
27	Goat Anti-Mouse IgG-HPR	Santa Cruz	sc-2005	
28	Lipofectamine 3000	ThermoFisher Scientific	L3000008	
29	RIPA Lysis and Extraction Buffer	ThermoFisher Scientific	89900	
30	Protease Inhibitor Cocktail Tablets	Millipore Sigma	118361700 01	
31	Nitrocellulose Membranes	Thermo Scientific	88024	
32	RIP-Assay Kit	MBL Life science	RN1001	

Supplementary Table 4. Reagents used in the study with source and catalog numbers.

*Assay ID, ** Concentration used
