# Supplementary material for: Functional characterization of the disease-associated CCL2 rs1024611G-rs13900T haplotype: The role of the RNA-binding protein HuR
Source: bioRxiv. 2023 Nov 2:2023.10.31.564937. Preprint. [Version 1] doi: 10.1101/2023.10.31.564937 (PMC10635030; doi:10.1101/2023.10.31.564937)
Supplement: Supplementary file 1 [file NIHPP2023.10.31.564937v1-supplement-1.pdf]

	CCL2 (rs13900 C/T) Control (42)
Wild allele [CC]	18 (42.8%)
Heterozygous [CT]	16 (38.09%)
Rare allele [TT]	8 (19.04%)
MAF	0.38
PIC	0.36
Heterozygosity	0.47
No of alleles	84
Hardy Weinberg *P*	0.459

Supplementary Table 1. Allele carriages and allele frequencies of rs13900 in healthy volunteers

	gDNA	Cytosol	Monosome	Polysome
D-1	0.975806	0.872946	0.873425	1.778429
D-2	1.034091	1.557778	1.441919	1.562622

Supplementary Table 2. Differential loading of rs13900 C>T onto polysome isolated from macrophages of heterozygous donors. C>T ratio < 1 indicates increased levels of the minor T allele relative to C allele. Result shows enrichment of rs13900T allele on polysomes.

Figure S1

A

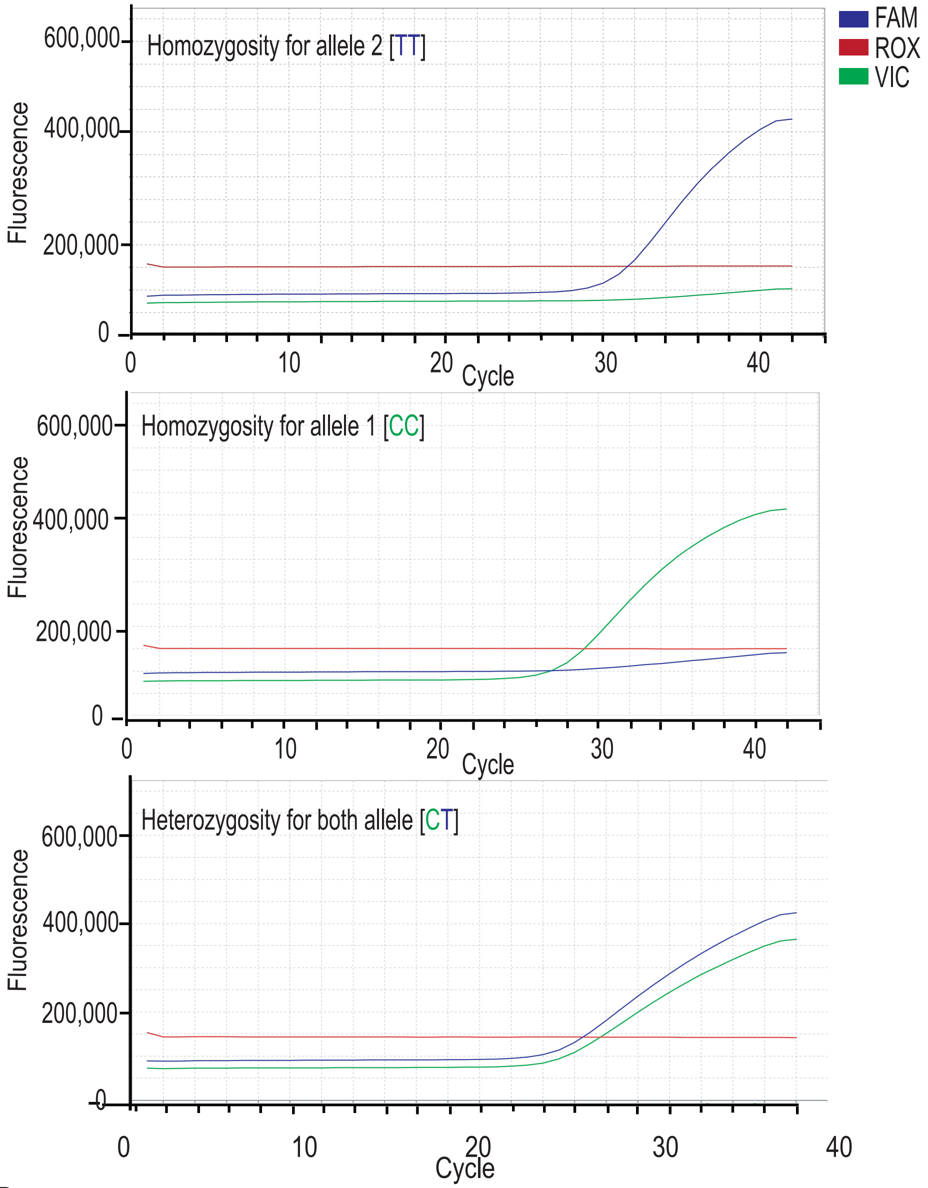
Homozygosity for allele 2 [TT]
FAM
ROX
VIC
Fluorescence
600,000
400,000
200,000
0
Cycle
0
10
20
30
40
Homozygosity for allele 1 [CC]
Heterozygosity for both allele [CT]

B

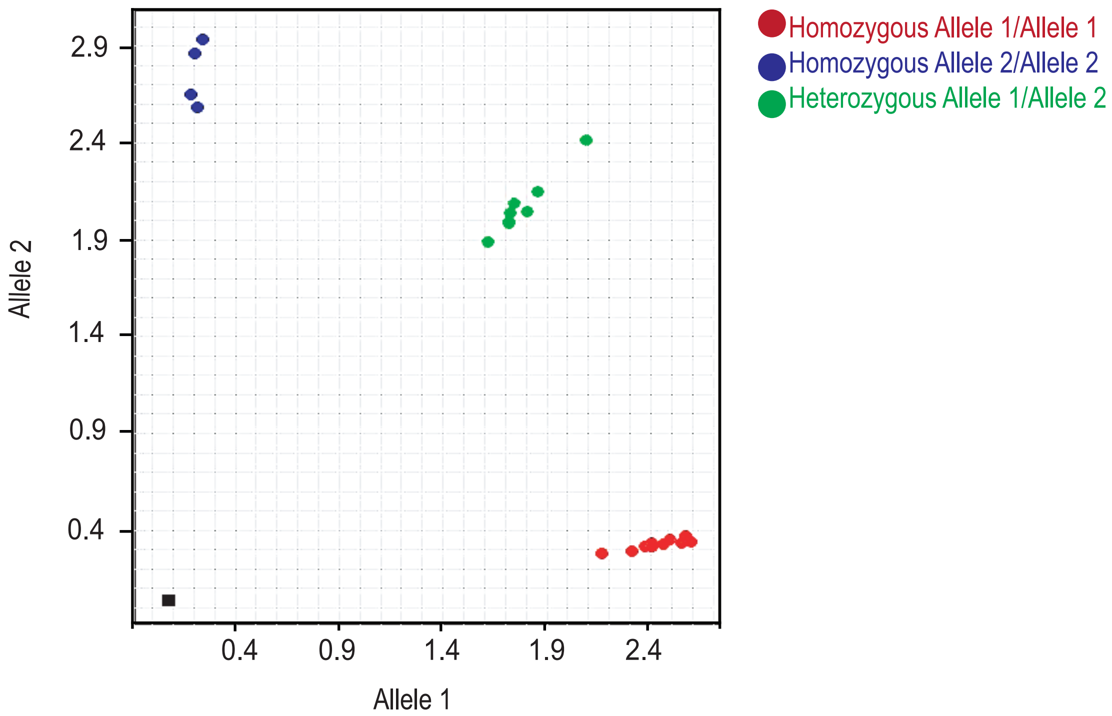
Homozygous Allele 1/Allele 1
Homozygous Allele 2/Allele 2
Heterozygous Allele 1/Allele 2
Allele 2
2.9
2.4
1.9
1.4
0.9
0.4
Allele 1

Figure S2

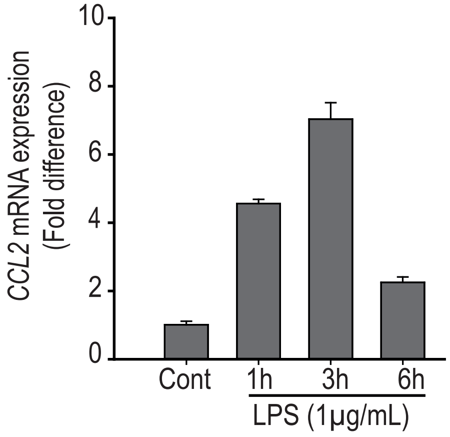

CCL2 mRNA expression
(Fold difference)
10
8
6
4
2
0
Cont
1h
3h
6h
LPS (1μg/mL)

Figure S3

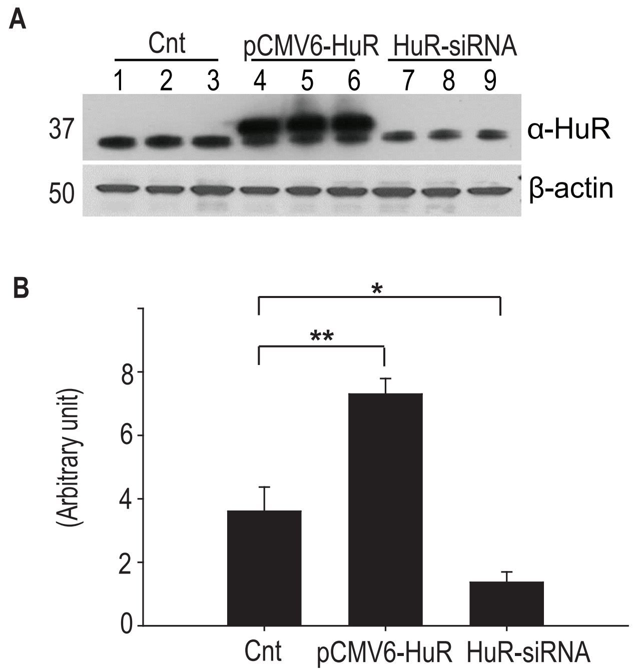
A
Cnt
pCMV6-HuR
HuR-siRNA
1 2 3 4 5 6 7 8 9
37
50
α-HuR
β-actin
B
(Arbitrary unit)
0
2
4
6
8
**
*
Cnt
pCMV6-HuR
HuR-siRNA

Figure S4

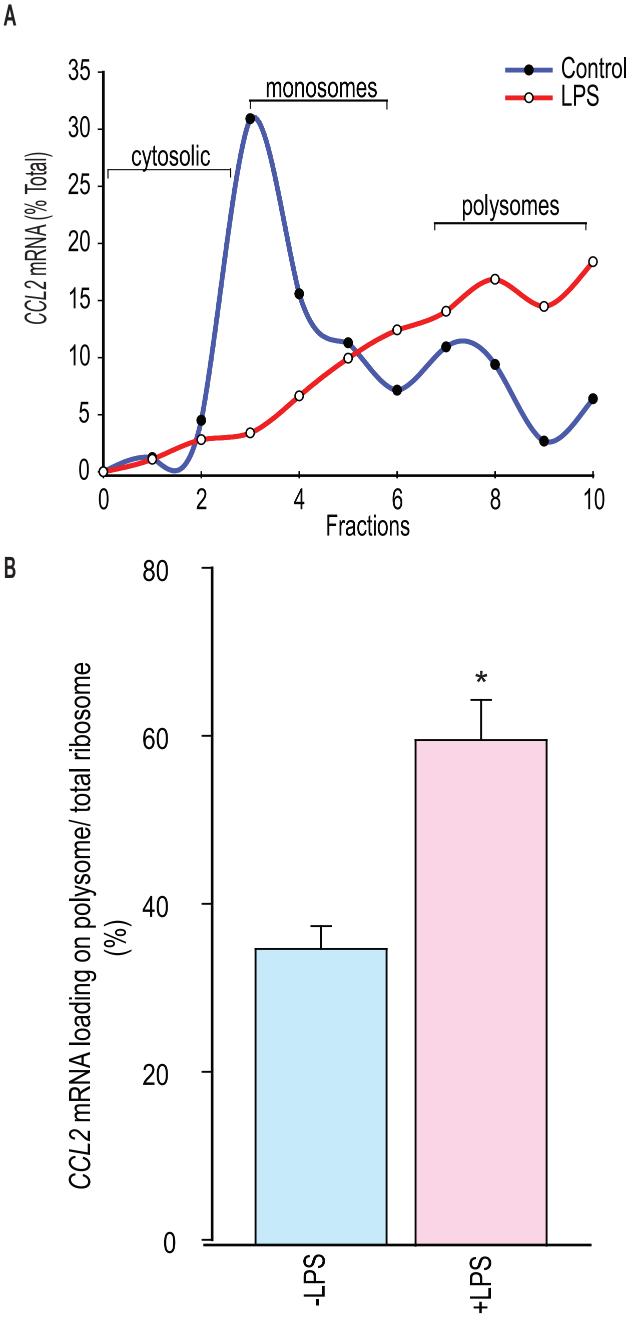
A
Control
LPS
35
30
25
20
15
10
5
0
monosomes
cytosolic
polysomes
CCL2 mRNA (% Total)
0
2
4
6
8
10
Fractions
B
80
60
40
20
0
*
CCL2 mRNA loading on polysome/ total ribosome (%)
-LPS
+LPS

Figure S5

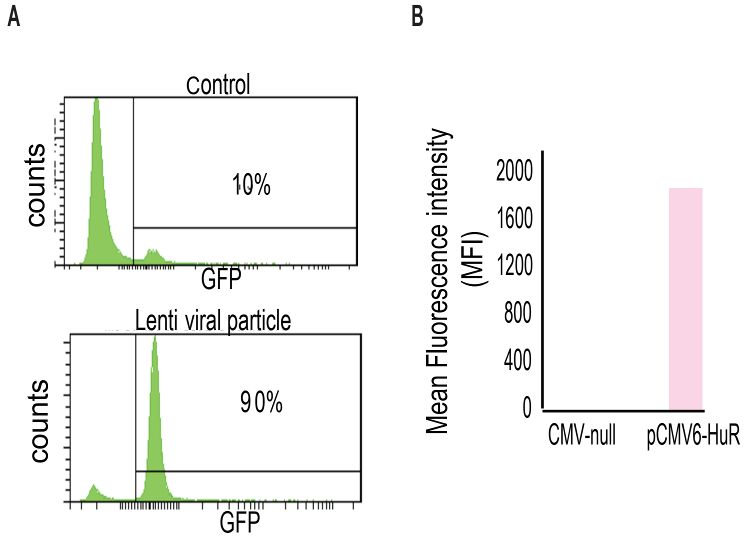

A
Control
counts
10%
GFP
Lenti viral particle
counts
90%
GFP
B
Mean Fluorescence intensity (MFI)
2000
1600
1200
800
400
0
CMV-null
pCMV6-HuR
